# Specific Vagus Nerve Lesion Have Distinctive Physiologic Mechanisms of Dysphagia

**DOI:** 10.3389/fneur.2019.01301

**Published:** 2019-12-12

**Authors:** François D. H. Gould, Andrew R. Lammers, Christopher J. Mayerl, Rebecca Z. German

**Affiliations:** ^1^Department of Cell Biology and Neuroscience, Rowan University School of Osteopathic Medicine, Stratford, NJ, United States; ^2^School of Health Sciences, Cleveland State University, Cleveland, OH, United States; ^3^Department of Anatomy and Neurobiology, NEOMED, Rootstown, OH, United States

**Keywords:** dysphagia, superior laryngeal nerve, recurrent laryngeal nerve, kinematics, animal model

## Abstract

Swallowing is complex at anatomical, functional, and neurological levels. The connections among these levels are poorly understood, yet they underpin mechanisms of swallowing pathology. The complexity of swallowing physiology means that multiple failure points may exist that lead to the same clinical diagnosis (e.g., aspiration). The superior laryngeal nerve (SLN) and the recurrent laryngeal nerve (RLN) are branches of the vagus that innervate different structures involved in swallowing. Although they have distinct sensory fields, lesion of either nerve is associated clinically with increased aspiration. We tested the hypothesis that despite increased aspiration in both case, oropharyngeal kinematic changes and their relationship to aspiration would be different in RLN and SLN lesioned infant pigs. We compared movements of the tongue and epiglottis in swallows before and after either RLN or SLN lesion. We rated swallows for airway protection. Posterior tongue ratio of safe swallows changed in RLN (*p* = 0.01) but not SLN lesioned animals. Unsafe swallows post lesion had different posterior tongue ratios in RLN and SLN lesioned animals. Duration of epiglottal inversion shortened after lesion in SLN animals (*p* = 0.02) but remained unchanged in RLN animals. Thus, although SLN and RLN lesion lead to the same clinical outcome (increased aspiration), the mechanisms of failure of airway protection are different, which suggests that effective therapies may be different with each injury. Understanding the specific pathophysiology of swallowing associated with specific neural insults will help develop targeted, disease appropriate treatments.

## Introduction

Many different types of neurological damage lead to similar outcomes of dysphagia or deglutive disorders, such as failure to propel the bolus, aspiration, or pharyngeal residue. For example, dysphagia is often associated with cortical injuries or conditions, such as stroke, amyotrophic lateral sclerosis, and cerebral palsy, and with midbrain conditions, such as Parkinson's disease (1). Dysphagia may also result from disorders that affect the myoneural junctions, such as myasthenia gravis. Similarly, injury to branches of the vagus nerve, whose axons synapse within the medulla and pons of the brainstem, also frequently result in dysphagia (2–4). Swallowing is a complex, coordinated process involving 25 paired muscles, and five cranial nerves (sensory and motor) all coordinated by multiple brainstem and cortical loci (5). The temporal sequence of events is critical for the efficient passing of a bolus from the oral cavity into the esophagus, while simultaneously avoiding the airway (6, 7). It is this complex and integrated process that presents many different failure points leading to the same outcome. Yet understanding *where* and *how* that process is interrupted and compromised is necessary to design interventions to correct the outcome. Furthermore, the where and how is likely to be specific to the type of neurological insult, even if the outcome is not.

The laryngeal branches of the vagus nerve, which arise from or send fibers to several nuclei within the medulla of the brainstem, have different roles in swallowing. The superior laryngeal nerve's (SLN) sensory branch innervates the valleculae and structures superior to the vocal folds. The sensory signals from the SLN are carried in the vagus nerve to the nodose ganglion, and then synapse in the nucleus of the solitary tract. Clinical and experimental data demonstrate that stimulation of the SLN can initiate the swallow (5, 8–11). The recurrent laryngeal nerve (RLN) innervates the muscles of the vocal folds and laryngeal mucosa below the vocal folds. Motor neurons innervating the laryngeal muscles are located in the nucleus ambiguous. Sensory signals from the lower laryngeal mucosa travel to the nucleus of the solitary tract in the brainstem. The sensory portion of the RLN is known to be important in eliciting the cough reflex (12). Both clinically and experimentally, lesion of either the RLN or the SLN leads to increased aspiration (13–15).

Although insult to either SLN or RLN results in increased aspiration (16, 17), these two lesions impact the size and shape of the bolus in infant animal models differently (13, 18, 19). After surgical transection of the right SLN, the volume of the bolus was greater (13), although bolus size was not associated with increased aspiration. When the right RLN was cut, however, the bolus area was *smaller* post lesion, with evidence suggestive of an airway protection effect. Before RLN lesion, bolus size, and the success or failure of airway protection were unrelated. After lesion, however, smaller bolus size was associated with safer swallows (19).

Bolus size prior to a swallow is a result of oropharyngeal kinematics, and how the tongue processes food to form that bolus. Thus, the movements of the tongue are also part of the biomechanics that drive the swallow. How such kinematics differ between these two lesions is not clear, and how those kinematics produce the performance failure that is dysphagia is also unknown. The movements of the tongue are intricately tied to the kinematics of the hyoid bone and jaw during swallowing (20), as well as bolus volume (21). In this chapter, we present unpublished data comparing swallow duration and tongue movement during the swallow before and after transection of the SLN or RLN in infant pigs. Because bolus size prior to a swallow is a result of oropharyngeal kinematics, we hypothesize that tongue movement and swallow duration will also differ between SLN and RLN lesions.

## Methods

The data used for this paper were collected in two locations over 5 years, with resulting slight differences in protocols, which are described below where relevant. We will indicate where this impacts on our conclusions.

### Animals

Data on SLN lesions were collected at Johns Hopkins University School of Medicine on five infant pigs (Tom Morris farms, Reisterstown, MD) in 2010 and 2011. Pigs were delivered to the vivarium at 2–3 weeks of age and weighed 4–5 kg at the start of experiments. Pigs were trained to drink a pig milk replacement solution (Land O' Lakes Solustart, St. Paul, MN) five times a day via a bottle fitted with a modified nipple. All procedures were approved by the Johns Hopkins University IACUC (protocol SW10M212). Some raw data from these pigs were generated for previous publications (13, 22), but a fifth pig that had not hitherto been digitized was added for this paper.

Data on the RLN lesions was collected at Northeast Ohio Medical University (NEOMED) in 2014 on six infant pigs (Michael Fanning Farm, Howe, IN) aged 5–14 days on arrival. Pigs were trained to feed on the same milk replacer and bottle as the pigs used for superior laryngeal nerve studies. All procedures were approved by the NEOMED IACUC committee (protocol 13–011). Data presented in this paper were originally generated for a previous publication (23).

### Procedures and Surgeries

Pigs in both groups underwent similar procedures as the studies were designed to mirror each other. Under isoflurane anesthesia (2–5%), radiopaque markers were implanted intraorally into the tongue, soft palate, and the gingiva under the hard palate. A radiopaque hemoclip (Weck Ligation Solutions, NC) was attached to the tip of the epiglottis. Subsequently, pigs underwent surgery under full aseptic conditions to implant EMG electrodes and identify the relevant nerve on the right side (RLN or SLN). Pigs were intubated and maintained in a stable plane of anesthesia throughout (0.5–3% isoflurane). During the surgery, radiopaque markers were sutured to the hyoid bone and thyroid cartilage. Prior to implantation of electrodes, the appropriate nerve was located and marked with loosely tied suture. For the SLN lesion, the nerve was identified on the right side originating from the vagus nerve in the carotid sheath and followed caudally on the surface of the thyrohyoid membrane. Two pieces of loose suture were tied close to where the SLN emerged from the carotid sheath in the carotid triangle (13, 22). For the RLN, the recurrent portion nerve was identified on the right side coursing lateral and dorsal to the trachea, deep to the infrahyoid muscles. The nerve was traced until it was seen entering the larynx by passing deep and dorsal to the cricothyroid muscle under the thyroid shield. In its long section close to the trachea, the nerve was loosely tied with suture for future identification. After nerve identification, marking of the hyoid and thyroid and EMG electrode implantation, the incision was closed with suture. One to four hours after surgical recovery, animals were taken to the fluoroscopy suite for recording as detailed below. As these recordings were pre-lesion, they constituted control recordings for each animal.

Thirty-six to seventy-two hours after the initial surgery, animals underwent a second surgery. A second incision was made on the right side lateral to the initial incision above the area where the nerve had been located. Using the suture placed around the nerve, the target nerve in each case (SLN, including both internal and external branches, or RLN) was located, then ligated in two places and fully transected with scissors, and the ends displaced to prevent regrowth. Data collected after this surgery were postlesion data. Animals received analgesics and antibiotics from before the first surgery and continuously throughout the experiments as needed.

### Videofluoroscopy

Pigs were recorded in lateral view feeding unrestrained in a plexiglass box. The SLN pre- and post-lesion pigs were filmed at either 60 frames per second (Allura FD20, Philips Healthcare, Best, The Netherlands) or 30 frames per second (Infinix-I, Toshiba Corporation, Tokyo, Japan). The RLN pre- and post-lesion pigs were filmed on a modified C-arm (GE9400 C-Arm) connected to a high speed (100 frames per second) digital video camera (XC 1M digital video camera, Xcitex, Cambridge, MA). Pigs were filmed drinking milk mixed with barium to visualize swallows. Videos were saved as AVI files for subsequent analysis.

### Scoring of Swallows for Airway Protection

Control and lesion swallows for both groups of pigs were assessed for effectiveness of airway protection using the Infant Mammalian Penetration Aspiration Scale (IMPAS) (24). This validated, ordinal ranking scale scores infant liquid swallows for penetration and aspiration from 1 (no milk enters the airway at any point) to 7 (silent aspiration: milk passes below the vocal folds and no attempt is made to clear the milk) (Table 1). Swallows were scored by individuals trained together in using the IMPAS scale at NEOMED. Training followed the protocol described in Holman et al. (24) to ensure agreement between raters.

**Table 1:** Summary of the IMPAS scale.

**Score**	**What happens**
1	Normal swallow
2	Some penetration that is cleared during the swallow
3	Some penetration that is not cleared during the swallow
4	A lot of penetration that is not cleared during the swallow
5	Aspiration with a successful attempt to clear
6	Aspiration with an unsuccessful attempt to clear
7	Aspiration with no attempt to clear

### Digitizing of Markers

One hundred and seventy-seven control and lesion swallows were identified from the SLN lesion pigs, and 113 control and lesion swallows for the RLN pigs. X and Y coordinates of the tongue, epiglottis, hard and soft palate, hyoid, and thyroid cartilage markers were digitized using either manual or automated marker tracking in specialized software (ProAnalyst, Xcitex, MA) for all frames of each swallow. Marker coordinates were then translated, rotated, and scaled to the two markers in the hard palate so that the hard palate became the horizontal axis with the anterior hard palate marker as the origin of the reference system. This transformation ensures that movements of the other markers are now described relative to a fixed hard palate, which removes the effect of full head motions during feeding (23, 25).

After rotation, two swallow-specific kinematic metrics were calculated from the digitized X/Y coordinates. *Duration of epiglottal flip* was calculated as the time between when the epiglottis begins its caudal movement to when it returns to its resting position, which is considered to be equivalent to the duration of the pharyngeal swallow (22). *Posterior tongue ratio* was calculated as the ratio of the distance traveled by the posterior tongue marker from the beginning of epiglottal movement to the time when the epiglottis reaches its most caudal point to the total distance traveled by the posterior tongue marker throughout the duration of epiglottal flip (23).

### Analysis

Because of the low number of aspiration events seen in the SLN sample, we combined IMPAS scores of 3, 4, and 7 into a single category for subsequent analysis. No scores of 5 or 6 were observed. Furthermore, because of the low number of more serious penetration and aspiration levels in control animals (scores 3–7), we subdivided our test of the effect of lesion on kinematics into two subtests, following Gould et al. (23). First, we tested the hypothesis that the impact of RLN and SLN lesion on posterior tongue ratio in safe (IMPAS 1 and 2) swallows would differ, using a mixed model with nerve (SLN or RLN), condition (control or lesion), and airway protection outcome (1 and 2) as fixed factors and individual as a random factor. Secondly, we tested the hypothesis that the relationship between swallow safety and posterior tongue ratio in post lesion swallows only would be different for RLN and SLN groups. Once again we used a mixed model, with airway protection outcome (safe swallows with IMPAS scores of 1 or 2 vs. unsafe swallows with IMPAS scores of 3–7) and nerve group (SLN and RLN) as fixed factors and individual as a random factor. Because of the differences in the treatment of the SLN and RLN groups of pigs, we nested condition and airway protection within nerve group in all analyses. Where significant main effects were observed, we used *post hoc* pairwise Tukey tests on the least squares means to determine what the significant differences were. We carried out an identical set of analyses on swallow duration (i.e., duration of epiglottal flip).

To test our hypothesis that epiglottal flip duration would differ between RLN and SLN lesion, we used a mixed model with condition (control or lesion) nested within nerve (SLN or RLN), and individual as a random factor. All analyses were done in R (26), using the packages lme4, lmerTest, and emmeans. We reported the variance of the random factor as an absolute and a percentage of total variation in the model for each test.

## Results

### The Effect of Nerve Lesion on Posterior Tongue Ratio for Safe Swallows Is Different for RLN and SLN Lesions

**Figure 1:**
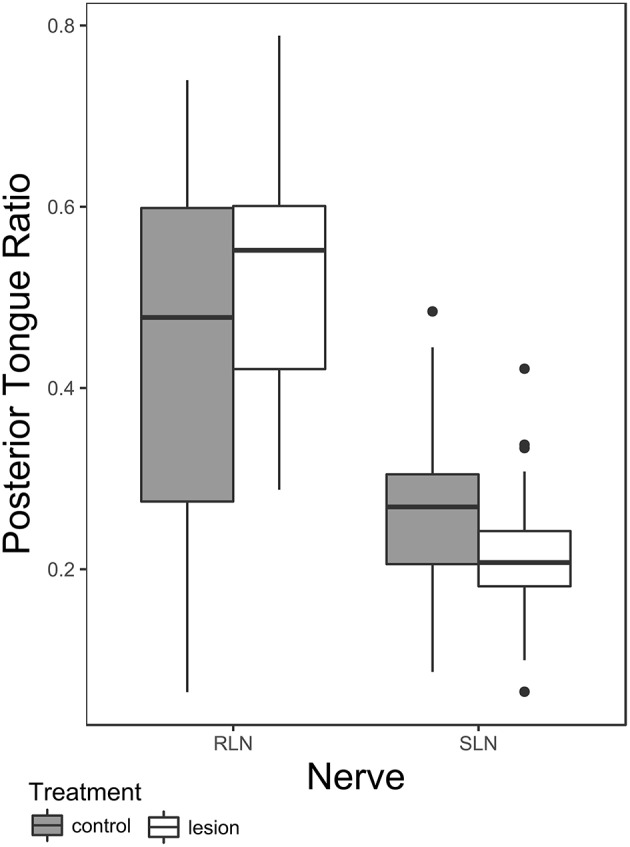
Box plot comparing posterior tongue ratio pre- and post lesion in SLN or RLN lesioned animals. Note that the data presented here includes only safe swallows (i.e., IMPAS 1 and 2). The box represents 50% of the data, with the line near the middle representing the median. Whiskers extend to 1.5 times the interquartile range from the median. Dots are outliers.

There is a significant main effect of nerve group (SLN vs. RLN) and the nerve group-condition interaction (pre- vs. post-lesion for each nerve group) on posterior tongue ratio in safe swallows (Table 2). *Post hoc* pairwise tests on the least squares means of treatment within nerve group reveal a significant effect of RLN lesion on posterior tongue ratio [t ratio (138.53) = −2.577, *p* = 0.01], but no effect of SLN lesion [t ratio (135.88) = 1.897, *p* = 0.06]. Furthermore, the polarity of change between the pre and post lesion means is different between the two groups: posterior tongue ratio tends to increase in RLN lesion (pre lesion mean 0.43 ± 0.037 standard error (SE); post lesion mean 0.51 ± 0.039 SE), and tends to decrease in SLN lesion (pre lesion mean 0.27 ± 0.05 SE; post lesion mean 0.21 ± 0.05 SE) (Figure 1). The controls for the two treatments are different, reflecting the significant nerve group factor. The variance of the individual factor was 0.006, representing 9.31% of total variation in the model.

**Table 2:** Results of mixed model analysis of the effects of RLN vs. SLN lesion on posterior tongue ratio in safe swallows (IMPAS 1 or 2).

**Factor**	**F (numerator df, denominator df)**	***P*-value**
**Nerve group**	**7.85 (1, 10.17)**	**0.018**
**Nerve group: condition**	**5.16 (2, 137.2)**	**0.007**
Nerve group:IMPAS	0.02 (2, 139.79)	0.985
Nerve group:condition:IMPAS	0.67 (2, 136.64)	0.513

*Bold indicates statistically significant effects*.

### Post Lesion, the Difference in Posterior Tongue Ratio in Safe and Unsafe Swallows Is Not the Same in SLN vs. RLN Lesioned Animals

**Figure 2:**
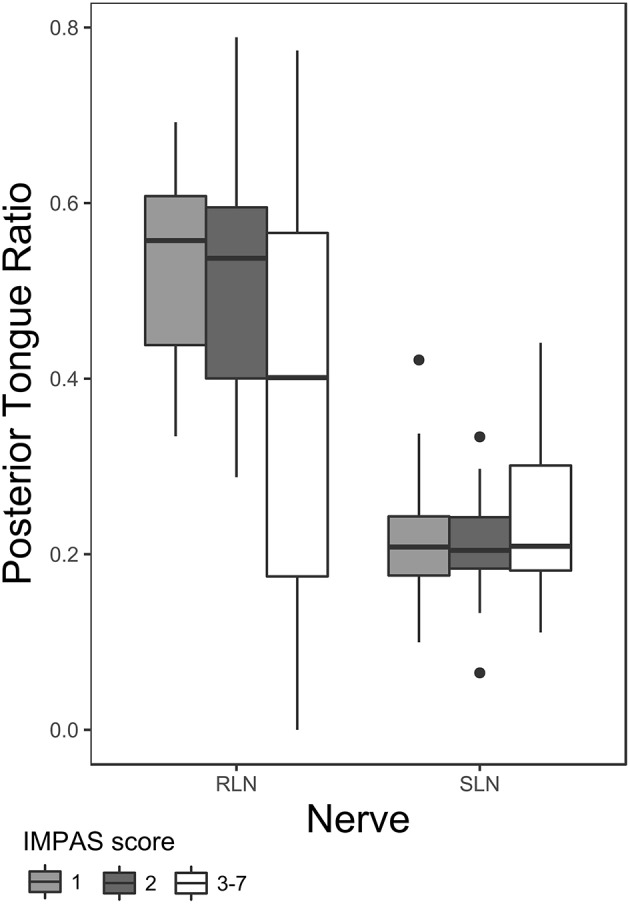
Box plot of posterior tongue ratio for IMPAS 1, 2, and 3–7 in post lesion swallows of RLN and SLN lesioned animals. The box represents 50% of the data, with the line near the middle representing the median. Whiskers extend to 1.5 times the interquartile range from the median. Dots are outliers.

Posterior tongue ratio differed significantly between SLN and RLN lesions. After the lesion surgery, the posterior tongue ratio was higher in the RLN lesion group compared with the SLN lesion group. Furthermore, nerve group—airway protection interaction on posterior tongue ratio in lesioned animals was also significant (Table 3 and Figure 2). *Post hoc* pairwise tests within nerve group indicate a significant difference between swallows with an IMPAS score 1 (mean 0.51 ± 0.051 SE) and swallows with IMPAS score of 3 to 7 (mean 0.38 ± 0.042 SE) in RLN lesioned animals [t ratio (109.63) = 2.657, *p* = 0.044] but in SLN lesioned animals there is no significant difference in posterior tongue ratio between swallows with different airway protection outcomes. The variance of the individual random factor was 0.006, representing 20% of the variation in the total model.

**Table 3:** Results of mixed model analysis of the effect of RLN and SLN lesion on posterior tongue ratio and airway protection in lesion swallows only.

**Factor**	**F (numerator df, denominator df)**	***P*-value**
**Nerve group**	**13.57 (1, 16.37)**	**0.002**
**Nerve group: IMPAS**	**2.52 (4, 115.2)**	**0.045**

*Bold indicates statistically significant effects*.

### Epiglottal Flip Duration Shortens in SLN Lesioned Swallows, Not RLN Lesioned Swallows

**Figure 3:**
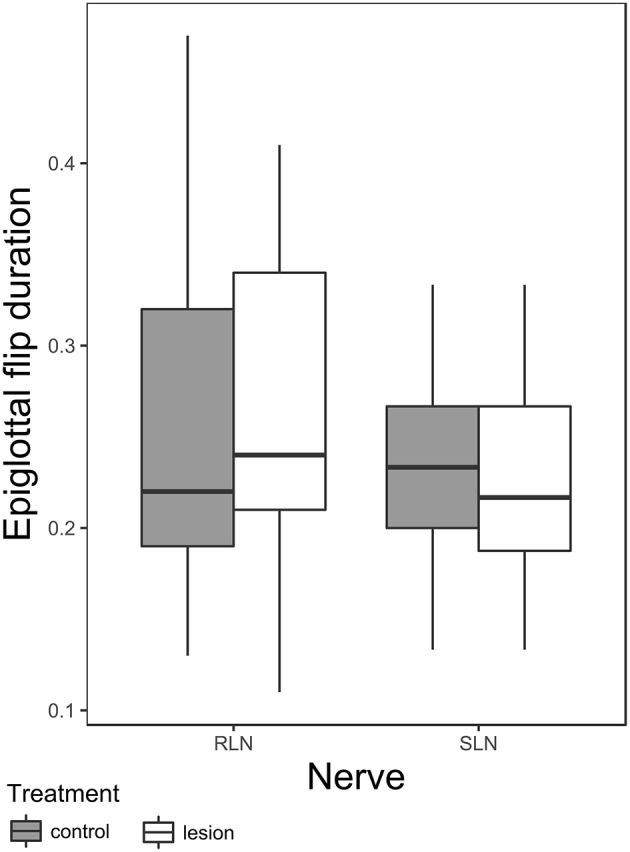
Box plot of duration of epiglottal flip in RLN and SLN lesioned animals. The box represents 50% of the data, with the line near the middle representing the median. Whiskers extend to 1.5 times the interquartile range from the median. Dots are outliers.

There is a significant effect of nerve group-condition interaction on epiglottal flip duration (Table 4 and Figure 3). *Post hoc* tests within nerve group reveal a significant shortening of epiglottal flip duration in SLN lesioned animals [t-ratio (277.08) = 2.343, *p* = 0.02, pre lesion mean 0.24 ± 0.022 SE, post lesion mean 0.23 ± 0.022 SE], but no significant effect in RLN lesioned animals. The variance of the individual random factor was 0.003, representing 56.52% of the variation in the sample.

**Table 4:** Results of mixed model analysis of the effect of RLN and SLN lesion on duration of epiglottal flip.

**Factor**	**F (numerator df, denominator df)**	***P*-value**
Nerve group	0.016 (1, 11.03)	0.9
**Nerve group: condition**	**3.32 (2, 277.15)**	**0.037**

*Bold indicates statistically significant effects*.

## Discussion

### Despite Both Resulting in Increased Aspiration, RLN and SLN Lesions Have Different Effects on Swallowing Physiology

The results of this study show that similar patterns to what is found with bolus size also apply to swallowing mechanics and kinematics, namely movement of the posterior tongue and duration of epiglottal inversion. Our results agree with previous studies that documented that RLN and SLN lesion affected bolus size differently (13, 19). Furthermore, how all these swallow parameters relate to airway protection deficits after lesion of either nerve also differs, indicating the specific mechanism of aspiration in SLN and RLN lesion is different.

Based on the results of this study, and the work by Ding et al. (13) and Gould et al. (19), we can propose hypotheses associated with the specific neurological functions of the SLN and RLN that could explain these differences. The increase in bolus size following SLN lesion matches the SLN's role as a swallow trigger (5, 9–11). Unilateral SLN lesion will therefore result in decreased sensitivity to the swallow stimulus by reducing the pool of available sensory receptors, thus requiring a bigger bolus to trigger the swallow. Large boluses are generally associated with longer swallow transit times (27); while our data indicated that swallow duration after SLN lesion was *shorter*, the study by Ding et al. (22) found *longer* swallow durations after transecting the SLN. Our own contradictory result around swallow duration likely resulted from using some different individuals in the analysis. Such variation was consistently found in all of the RLN lesion studies (14, 19, 23) as well as other studies of sensory disruption (25). Thus, in these animals, variation in response to insult seems to characterize dysphagia.

However, although the SLN is important in triggering the swallow response, its sensitivity is highly modulated by oral sensation arising from the trigeminal and glossopharyngeal nerves. For example, palatal anesthesia modifies both swallowing kinematics and airway protection (24), and oral stimulation through rhythmic delivery affects bolus volume that trigger swallowing (28). Thus, the impact of unilateral SLN lesion on swallowing function, bolus volume, and airway protection in otherwise neurologically intact animals will be modified by other oral sensory pathways. Further studies looking at the impact of different oral sensory stimuli on animals with SLN lesion will be necessary to clarify these relationships.

High levels of inter-individual variability itself has implications for neural control (29). Posterior tongue ratio does not seem to change pre- to post-lesion in the SLN lesioned animals, nor to differ between safe and unsafe swallows. This suggests that the relative timing of the movements of different structures during the early stages of the pharyngeal swallow is not affected by SLN lesion. Indeed, none of the parameters examined in this study are strongly associated with airway protection, suggesting that the mechanism of aspiration after SLN lesion is related to physiological parameters not captured in this study, and are more likely linked to increased bolus sizes (13). These results suggest that the larger bolus, coupled with the sensory deficit resulting from unilateral SLN transection, is simply more difficult to control. This hypothesis requires further testing.

In contrast, the pattern for the RLN suggests a more subtle, but more widespread disruption of swallowing coordination (6). Bolus volume is on average smaller post lesion, and here larger boluses are associated with failure of airway protection after RLN lesion (19). Furthermore, there is a change in the relative timing of tongue movement post lesion, which is also associated with airway protection, particularly to achieve safe swallows. Thus, in the RLN lesioned animals, we see changes in efficiency (as safe swallows require smaller boluses) tied to changes in the relative timing of movements in the early part of the pharyngeal swallow. Because these changes are related to airway protection outcomes, it seems more likely that the mechanism of aspiration is related to kinematics and bolus formation early in the swallow. Indeed, work on the effects of RLN lesion on patterns of muscle activation, both duration and timing, in swallowing has shown changes in the timing of muscle contraction for muscles located in the floor of the mouth, and which are active early in the swallow sequence before bolus formation (30). However, a complete comparison requires similar data for the SLN lesion animals.

Further supporting the idea that the mechanism of aspiration in RLN and SLN lesion animals are different, the time of aspiration in each case differs. In SLN lesioned animals, aspiration tends to occur *during* the swallow (22). However, in RLN lesioned animals aspiration can occur either during or *after* the swallow (14). Here again, differences in the details of the timing of events suggest differences in underlying mechanism of airway protection failure.

These hypotheses for the neural origin of kinematic differences between RLN and SLN lesioned pigs also suggest that different interventions may be effective to modify swallowing in each case. If the reduced sensitivity hypothesis is correct for explaining the patterns we see in the SLN lesioned animals, then either controlling bolus volume directly through regulated feeding, or increasing sensitivity of the valleculae by using other stimuli in conjunction with volume [i.e., capsaicin (31, 32)] are likely to be most effective for restoring normal swallow function. In the case of RLN lesion, however, restoring something like normal function is likely to involve interventions that harness sensory motor mechanisms that establish coordination between tongue and pharyngeal components of swallowing, such as entrained milk delivery (28) or motor learning (33–35).

### Limitations of This Study and Unknowns

The way in which the data presented in this study were collected means that some caution must be taken in interpreting these results. The two groups of pigs are different in their control kinematics. As these groups of animals were collected several years apart in two different locations, a number of factors may account for this. The SLN lesioned pigs were older by several days then the RLN lesioned pigs. Pigs reach weaning age in about 25 days, and show maturation of certain feeding behaviors in that time (36, 37). In particular, the (younger) RLN pigs were considerably variable in all studies (14, 19, 23). Thus, the younger age of the RLN pigs may account for the greater variation in control kinematics seen in this study. The specific statistical model used here, which controls for individual variation and, by using nesting, does not pool the RLN and SLN control data, goes some way to mitigating this variation. As a proportion of total variance in the model, the importance of individual variation varies depending on what aspect of oropharyngeal function is being measured (tongue vs. epiglottal flip) and whether post-lesion only animals are being examined.

Technical differences, most notable the different maximum frame rate available for the SLN vs. the RLN pigs, also are a limitation. In particular this means that meaningful differences in kinematics between SLN and RLN pigs can only be measured to the precision of the slower frame rate (30–60 fps). Finally, airway protection, a major variable in our data set, is not experimenter controlled, but occurs *ad hoc* among animals. Rates of aspiration after RLN lesion are quite irregular among individuals (14). This variability limits our ability to look at fine grain differences between penetration and aspiration in this study, as the SLN lesioned pigs available for this work, while showing penetration, showed limited aspiration.

### A Better Etiology of Dysphagia Is Needed, One That Is Based on Functional Damage Effects Instead of Gross Outcomes

When discussing dysphagia in the context of neurological disorders, a significant list of conditions where dysphagia occurs is often presented (as indeed at the beginning of this paper). Knowing the prevalence and occurrence of dysphagia across different patient populations is important for epidemiology and public health. Yet, as this study shows, a diagnosis of dysphagia is insufficient for understanding the specific pathophysiology behind dysphagia in a given condition. Furthermore, the diagnostic endpoint (e.g., aspiration or residue), may result from very different processes going wrong within the swallow. On the other hand, when pathophysiology is identified and studied, for example tongue weakness in Parkinson's disease (38–40), its relationship to the diagnostic symptoms, again aspiration or residue, is often unclear. What is missing is a classification of dysphagia that accounts for the steps between neurological insult, pathophysiology, and diagnostic criterion, so that therapies can be developed that specifically target the disordered physiology.

Clinically, our work suggests that knowing how specific nerve lesions affect swallowing function in the context of aspiration could help inform treatment selection. As an example, the difference in bolus size between SLN and RLN lesioned suggests that bolus size restriction might be more useful in one type of lesion than another to prevent aspiration. Thus, when evaluating treatments, studies need to account as best as possible for the etiology of the dysphagia, as different etiologies may respond better or worse to different treatments. Further, the category “dysphagia,” or even “deglutitive disorders,” is very broad. Sorting out the specific, and possibly different, functional deficits in conditions that appear superficially similar is a critical first step in determining differences in the mechanisms that generate the pathophysiology. Understanding the mechanism, in turn, is critical for the design of effective interventions.

Our work on SLN and RLN lesions shows how a strictly experimental, systematic, basic science approach can provide the framework for beginning to understand these issues (6, 13, 14, 19, 22, 23). Animal model work is particularly well-suited to the hierarchical study of insult, pathophysiology, and disease (41). Indeed, the literature on animal models of neurological disorders increasingly combines attempts to model the complete disorder with targeted approaches seeking to reproduce a particular part of the neurological disorder to test hypotheses about pathophysiology (42). In our own work, we are currently working on steps to test interventions in infant pigs based on what we have learnt through the systematic analysis of the relationship between specific nerve lesions, pathophysiology, and dysphagic outcomes (43, 44). In order to build this systematic understanding of the relationship between specific neural insults, pathophysiology, and dysphagia in a context that will ultimately improve human health, collaborations between clinical researchers, human physiologists, and animal model workers are essential.

## Data Availability Statement

The datasets generated for this study are available on request to the corresponding author.

## Ethics Statement

The animal study was reviewed and approved by NEOMED IACUC.

## Author Contributions

FG, AL, and RG designed the study and performed experiments. FG and AL collected data and analyzed data. FG, AL, CM, and RG wrote and edited manuscript.

### Conflict of Interest

The authors declare that the research was conducted in the absence of any commercial or financial relationships that could be construed as a potential conflict of interest.

## References

[1] Logemann JA Evaluation and Treatment of Swallowing Disorders. Austin, TX: PRO-ED (1998). p. 432 10.1097/00020840-199812000-00008

[2] Jabbour J Martin T Beste D Robey T. Pediatric vocal fold immobility: natural history and the need for long-term follow-up. JAMA Otolaryngol Neck Surg. (2014) 140:428–33. 10.1001/jamaoto.2014.81 24626342

[3] Jafari S Prince RA Kim DY Paydarfar D. Sensory regulation of swallowing and airway protection: a role for the internal superior laryngeal nerve in humans. J Physiol. (2003) 550:287–304. 10.1113/jphysiol.2003.039966 12754311 PMC2343009

[4] Setlur J Hartnick CJ. Management of unilateral true vocal cord paralysis in children. Curr Opin Otolaryngol Head Neck Surg. (2012) 20:497–501. 10.1097/MOO.0b013e3283590b56 23150153

[5] Jean A. Brain stem control of swallowing: neuronal network and cellular mechanisms. Physiol Rev. (2001) 81:929–69. 10.1152/physrev.2001.81.2.929 11274347

[6] Gross A Ohlemacher J German R Gould FDH. LVC timing in infant pig swallowing and the effect of safe swallowing. Dysphagia. (2018) 33:51–62. 10.1007/s00455-017-9832-0 28780633 PMC7147992

[7] Young JL Macrae P Anderson C Taylor-Kamara I Humbert IA. The sequence of swallowing events during the chin-down posture. Am J Speech Lang Pathol. (2015) 24:659–70. 10.1044/2015_AJSLP-15-0004 26225454 PMC4698467

[8] Lang IM Medda BK Babaei A Shaker R. Role of peripheral reflexes in the initiation of the esophageal phase of swallowing. AJP Gastrointest Liver Physiol. (2014) 306:G728–37. 10.1007/978-94-017-8771-0 24557762 PMC3989705

[9] Sasaki CT Hundal JS Kim Y-H. Protective glottic closure: biomechanical effects of selective laryngeal denervation. Ann Otol Rhinol Laryngol. (2005) 114:271–5. 10.1177/000348940511400404 15895781

[10] Suzuki T Yoshihara M Sakai S Tsuji K Nagoya K Magara J. Effect of peripherally and cortically evoked swallows on jaw reflex responses in anesthetized rabbits. Brain Res. (2018) 1694:19–28. 10.1016/j.brainres.2018.05.002 29730058

[11] Takahashi K Shingai T Saito I Yamamura K Yamada Y Kitagawa J. Facilitation of the swallowing reflex with bilateral afferent input from the superior laryngeal nerve. Neurosci Lett. (2014) 562:50–3. 10.1016/j.neulet.2014.01.017 24462841

[12] Canning BJ. Anatomy and neurophysiology of the cough reflex: ACCP evidence-based clinical practice guidelines. Chest. (2006) 129:33S−47. 10.1378/chest.129.1_suppl.33S 16428690

[13] Ding P Campbell-Malone R Holman SD Lukasik SL Thexton AJ German RZ. The effect of unilateral superior laryngeal nerve lesion on swallowing threshold volume. Laryngoscope. (2013) 123:1942–7. 10.1002/lary.24051 23670486 PMC4307787

[14] Gould FDH Lammers AR Ohlemacher J Ballester A Fraley L Gross A. The physiologic impact of unilateral Recurrent Laryngeal Nerve (RLN) lesion on infant oropharyngeal and esophageal performance. Dysphagia. (2015) 30:714–22. 10.1007/s00455-015-9648-8 26285799 PMC4639401

[15] Varaldo E Ansaldo GL Mascherini M Cafiero F Minuto MN. Neurological complications in thyroid surgery: a surgical point of view on laryngeal nerves. Front Endocrinol. (2014) 5:108. 10.3389/fendo.2014.00108 25076936 PMC4097206

[16] Liu J Hai Y Kang N Chen X Zhang Y. Risk factors and preventative measures of early and persistent dysphagia after anterior cervical spine surgery: a systematic review. Eur Spine J. (2018) 27:1209–18. 10.1007/s00586-017-5311-4 28988275

[17] Tsujimura T Suzuki T Yoshihara M Sakai S Koshi N Ashiga H. Involvement of hypoglossal and recurrent laryngeal nerves on swallowing pressure. J Appl Physiol. (1985) 124:1148–54. 10.1152/japplphysiol.00944.2017 29357492

[18] Ding P Fung GS-K Lin M Holman SD German RZ. The effect of bilateral superior laryngeal nerve lesion on swallowing: a novel method to quantitate aspirated volume and pharyngeal threshold in videofluoroscopy. Dysphagia. (2014) 30:47–56. 10.1007/s00455-014-9572-3 25270532 PMC4351730

[19] Gould FDH Yglesias B Ohlemacher J German RZ. Pre-pharyngeal swallow effects of recurrent laryngeal nerve lesion on bolus shape and airway protection in an infant pig model. Dysphagia. (2017) 32:362–73. 10.1007/s00455-016-9762-2 27873091 PMC5420465

[20] Matsuo K Palmer JB. Kinematic linkage of the tongue, jaw, and hyoid during eating and speech. Arch Oral Biol. (2010) 55:325–31. 10.1016/j.archoralbio.2010.02.008 20236625 PMC2862248

[21] Tasko SM Kent RD Westbury JR. Variability in tongue movement kinematics during normal liquid swallowing. Dysphagia. (2002) 17:126–38. 10.1007/s00455-001-0112-6 11956838

[22] Ding P Campbell-Malone R Holman SD Lukasik SL Fukuhara T Gierbolini-Norat EM. Unilateral superior laryngeal nerve lesion in an animal model of *Dysphagia* and its effect on sucking and swallowing. Dysphagia. (2013) 28:404–12. 10.1007/s00455-013-9448-y 23417250 PMC3710536

[23] Gould FDH Ohlemacher J Lammers AR Gross A Ballester A Fraley L. Central nervous system integration of sensorimotor signals in oral and pharyngeal structures: oropharyngeal kinematics response to recurrent laryngeal nerve lesion. J Appl Physiol. (2016) 120:495–502. 10.1152/japplphysiol.00946.2015 26679618 PMC4773648

[24] Holman SD Campbell-Malone R Ding P Gierbolini-Norat EM Griffioen AM Inokuchi H. Development, reliability, and validation of an infant mammalian penetration–aspiration scale. Dysphagia. (2013) 28:178–87. 10.1007/s00455-012-9427-8 23129423 PMC3586779

[25] Holman SD Campbell-Malone R Ding P Gierbolini-Norat EM Lukasik SL Waranch DR. Swallowing kinematics and airway protection after palatal local anesthesia in infant pigs: swallowing after palatal anesthesia. Laryngoscope. (2014) 124:436–45. 10.1002/lary.24204 23686446 PMC4319539

[26] R Core Team. R: A Language and Environment for Statistical Computing. Vienna: R Foundation for Statistical Computing (2015). Available online at: http://www.R-project.org/ (accessed November 10, 2019).

[27] Shibata S Inamoto Y Saitoh E Kagaya H Aoyagi Y Ota K. The effect of bolus volume on laryngeal closure and UES opening in swallowing: kinematic analysis using 320-row area detector CT study. J Oral Rehabil. (2017) 44:974–81. 10.1111/joor.12573 28891595

[28] German RZ Crompton AW Owerkowicz T Thexton AJ. Volume and rate of milk delivery as determinants of swallowing in an infant model animal (*Sus scrofia*). Dysphagia. (2004) 19:147–54. 10.1007/s00455-004-0001-x 15383943

[29] Frigon A Chapter 7–interindividual variability and its implications for locomotor adaptation following peripheral nerve and/or spinal cord injury. Prog Brain Res. (2011) 188:101–18. 10.1016/B978-0-444-53825-3.00012-7 21333805

[30] DeLozier KR Gould FDH Ohlemacher J Thexton AJ German RZ. Impact of recurrent laryngeal nerve lesion on oropharyngeal muscle activity and sensorimotor integration in an infant pig model. J Appl Physiol. (2018) 125:159–66. 10.1152/japplphysiol.00963.2017 29648522 PMC6086969

[31] Kondo E Jinnouchi O Nakano S Ohnishi H Kawata I Okamoto H. Aural stimulation with capsaicin ointment improved swallowing function in elderly patients with dysphagia: a randomized, placebo-controlled, double-blind, comparative study. Clin Interv Aging. (2017) 12:1921–8. 10.2147/CIA.S138357 29180855 PMC5691921

[32] Shin S Shutoh N Tonai M Ogata N. The effect of capsaicin-containing food on the swallowing response. Dysphagia. (2016) 31:146–53. 10.1007/s00455-015-9668-4 26531834 PMC4824833

[33] Humbert IA Christopherson H Lokhande A German R Gonzalez-Fernandez M Celnik P. Human hyolaryngeal movements show adaptive motor learning during swallowing. Dysphagia. (2013) 28:139–45. 10.1007/s00455-012-9422-0 22926828 PMC3530020

[34] Humbert IA German RZ. New directions for understanding neural control in swallowing: the potential and promise of motor learning. Dysphagia. (2013) 28:1–10. 10.1007/s00455-012-9432-y 23192633 PMC3895459

[35] Park SH Casamento-Moran A Singer ML Ernster AE Yacoubi B Humbert IA. Integration of visual feedback and motor learning: corticospinal vs. corticobulbar pathway. Hum Mov Sci. (2018) 58:88–96. 10.1016/j.humov.2018.01.002 29353095

[36] Ballester A Gould FDH Bond L Stricklen B Ohlemacher J Gross A. Maturation of the coordination between respiration and deglutition with and without recurrent laryngeal nerve lesion in an animal model. Dysphagia. (2018) 33:627–35. 10.1007/s00455-018-9881-z 29476275 PMC6108960

[37] Crompton AW German RZ Thexton AJ. Development of the movement of the epiglottis in infant and juvenile pigs. Zoology. (2008) 111:339–49. 10.1016/j.zool.2007.10.002 18387794 PMC2574016

[38] Ciucci MR Russell JA Schaser AJ Doll EJ Vinney LM Connor NP. Tongue force and timing deficits in a rat model of Parkinson disease. Behav Brain Res. (2011) 222:315–20. 10.1016/j.bbr.2011.03.057 21459116 PMC3113690

[39] De Letter M Santens P Van Borsel J. The effects of levodopa on tongue strength and endurance in patients with Parkinson's disease. Acta Neurol Belg. (2003) 103:35–8. 12704981

[40] Umemoto G Tsuboi Y Kitashima A Furuya H Kikuta T. Impaired food transportation in Parkinson's disease related to lingual bradykinesia. Dysphagia. (2011) 26:250–5. 10.1007/s00455-010-9296-y 20803220

[41] German RZ Crompton AW Gould FDH Thexton AJ. Animal models for *Dysphagia* studies: what have we learnt so far. Dysphagia. (2017) 32:73–7. 10.1007/s00455-016-9778-7 28132098 PMC5545751

[42] Lind LA Murphy ER Lever TE Nichols NL. Hypoglossal motor neuron death via Intralingual CTB-saporin (CTB-SAP) injections mimic aspects of Amyotrophic Lateral Sclerosis (ALS) related to dysphagia. Neuroscience. (2018) 390:303–16. 10.1016/j.neuroscience.2018.08.026 30179644 PMC6168367

[43] Gould FDH Gross A German RZ Richardson JR. Evidence of oropharyngeal dysfunction in feeding in the rat rotenone model of Parkinson's Disease. Parkinson's Dis. (2018) 2018:6537072. 10.1155/2018/6537072 29713446 PMC5866867

[44] Mayerl CJ Gould FDH Bond LE Stricklen BM Buddington RK German RZ. Preterm birth disrupts the development of feeding and breathing coordination. J Appl Physiol. (2019) 126:1681–6. 10.1152/japplphysiol.00101.2019 31018743 PMC6620663

